# Quality Control Standards for Batch Effect Evaluation and Correction in Mass Spectrometry Imaging

**DOI:** 10.1021/acs.analchem.5c02020

**Published:** 2025-05-12

**Authors:** Luojiao Huang, Yaejin Kim, Benjamin Balluff, Berta Cillero-Pastor

**Affiliations:** † Cell Biology-Inspired Tissue Engineering, Institute for Technology-Inspired Regenerative Medicine, 5211 Maastricht University, 6229ER Maastricht, Netherlands; ‡ Maastricht MultiModal Molecular Imaging Institute, Division of Imaging Mass Spectrometry, Maastricht University, 6229ER Maastricht, Netherlands

## Abstract

Matrix-assisted laser desorption/ionization mass spectrometry imaging (MALDI-MSI) allows spatial molecular profiling. Despite many successful applications, an appropriate control of technical variations is still lacking for result reproducibility assessment and for maximizing the MSI data quality. To address this, we introduce a novel quality control standard (QCS) design and data analysis pipeline accounting for variability due to sample preparation and instrument performance. Firstly, we created a tissue mimicking QCS consisting of propranolol in a gelatin matrix. We showed that this QCS mimics ion suppression of propranolol in the tissue. Next, a three-day batch experiment demonstrated the QCS’s performance to longitudinal technical variations, establishing it as an effective indicator of batch effects. Then three computational approaches for batch effect correction were applied for the first time to MALDI-MSI data, leading to a significant reduction of QCS variation and to improved sample clustering by using multivariate principal component analysis. Altogether, we offer the designed QCS in combination with a data correction pipeline for MALDI-MSI users for batch effect evaluation and correction.

## Graphical abstract

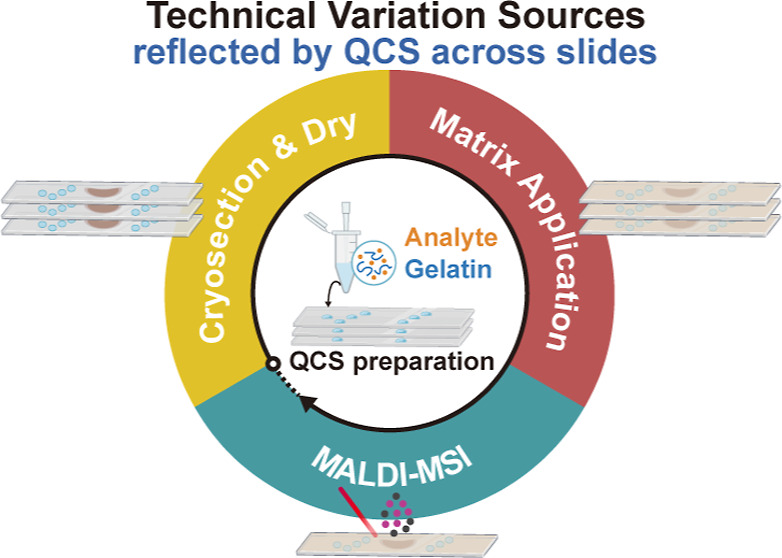

Omics analyses have been broadly used in biomedical research due to their excellent advantages in collecting biomolecular information in a high-throughput manner, thereby helping in the understanding of biological processes and assisting on biomarker discovery. In recent years, spatial omics techniques have gained attention since they add information on the location of certain molecular changes.^1^

One of these is the family of mass spectrometry imaging (MSI) techniques. These are especially regarded as label-free tools that utilize a laser, charged droplets, or an energetic ion beam to spatially desorb and ionize molecules from typically biological organs and tissues,^2,3^ whole animal sections,^4,5^ cells,^6,7^ or 3D models.^8^ Matrix-assisted laser desorption/ionization (MALDI) is the most popular MSI technique since it can, due to the use of an organic matrix as intermediate, map analytes including but not limited to proteins, peptides, glycans, lipids, metabolites, and drug compounds. The spatial molecular profiles collected by MSI from tissue regions are usually analyzed by multivariate methods for correlation with tissue types, disease stages, or treatment outcomes.^9,10^ Predictive classifiers can be trained and tested for cancer diagnosis, prognosis, and tumor classification for precision treatment.^11^ This holds great potential for improving pathological diagnostics.

However, and similar to other omics technologies, one major bottleneck that can compromise the implementation of MALDI-MSI for clinical use can be technical variation.^12^ A systematic source of technical variation that affects a larger number of samples (the “batch”) in the same way is termed “batch effect”.^13^

Many artifacts can lead to a batch effect in MSI, ranging from sample collection and preparation to data acquisition. Recently, these causes have been categorized into five different levels: on a pixel, section, slide, time, and location (center/laboratory) level.^13^ On all these levels, a batch effect can, if large enough, either mask the desired detection of a biological effect or lead to false-positive correlations.^13^ To mitigate this impact as much as possible, several methodologies can be taken into consideration during the stages of experimental design and data analysis. To reduce false-positive results, randomization and blocking can effectively reduce any systematic bias, especially those time-dependent variations in big batches. To minimize false-negative results, outlier detection and removal can increase the sensitivity to biology-caused variation.^13,14^ Also, data normalization increases sample comparability by bringing all samples to the same scale, which can mitigate the batch effect to a certain extent. Common approaches include total ion count (TIC) normalization, median normalization, and internal standard (IS) normalization, if applicable.^15^ In addition to these mass spectrometry-originated normalization techniques, several generic data processing methods have been developed to correct for batch effects of omics datasets. These methods include quality controls (QCs)-based methods (RLSC,^16^ SVRC,^17^ and SERRF^18^), location-scale methods (Combat^19^ and Combat-Seq^20^), matrix factorization methods (ICA,^21^ WaveICA,^22^ SVD,^23^ and EigenMS^24^), and deep neural networking methods (NormAE^25^). To help determine the benefit of different computational batch effect correction methods, the use of a quality control reference sample can play a helpful role in choosing the right algorithm and parameters. This strategy of combining quality control reference samples with data correction packages has never been applied to MALDI-MSI.

Introducing quality control standards (QCSs) thus becomes indispensable for MSI to help monitor, evaluate, or correct batch effects. QCSs should ideally reflect the technical variation across the entire experimental workflow while also assessing each step independently. To achieve this, QCSs should be incorporated at the earliest possible stage of the workflow, remaining alongside the sample until the end and providing insights into specific aspects where potential issues might arise. In LC–MS omics experiments, pooling and aliquoting of biological samples can function as quality control references. On the one hand, it estimates the technical variations introduced at steps of sample extraction, preparation, and instrument performance.^26,27^ On the other hand, it also evaluates the correction efficiency. However, this is more difficult in MSI since pooling and aliquoting are not possible. Nevertheless, alternative strategies have been proposed to establish appropriate QCS for MSI. These include the use of single standards and other bio-tissue-based controls: for single standards, serially diluted bovine serum albumin has been used as an external control on a target plate to assess the technical variation of instrument performance and determine the detection limit prior to each batch.^28^ Zhang et al. have employed lipid standards, homogeneously deposited on a blank slide, to evaluate method reproducibility and mass accuracy prior to single-cell MS imaging.^29^ Besides, cytochrome *c* has been applied to monitor the efficiency of trypsin digestion for peptide MSI.^30^ Overall, quality checks via a single standard approach can be performed per day or slide, with per-slide checks being more efficient for outlier detection.

Alternative tissue-based controls, such as calibrant deposition on intact tissue^31^ or standards spiked into homogenized tissue,^32^ have also been reported. While the former has only a limited focus on the calibration of mass accuracy,^31^ the latter approach allows for evaluating response linearity and variability with the spiked standards and has been mainly applied in drug quantification.^32^ Given good tissue homogeneity, homogeneous tissues of human liver and gastrointestinal stromal tumor tissue were used to score a given MALDI-MSI method, in terms of homogeneity of on-slide tissue processing and MALDI-MSI analysis as well as for quantification of inter-day repeatability.^33^ Focused on the variability of endogenous molecules, recent work used homogenized egg white as a quality control for peptide and N-glycan MALDI-MSI. This approach could evaluate digestion efficiency, mass accuracy, and signal variability over slides or batches or across data from multiple sites.^34^

However, the downside of the so far proposed solutions lies either in the laborious preparation or in the variability of the biological sources. To overcome these challenges, we propose the controlled creation of QCS based on tissue mimicking materials. Extracellular matrix (ECM) functions as a major protein intricate component of biological tissues and thus affects the ionization efficiency in MS. Therefore, gelatin can be regarded as an appropriate substitute material due to its origin from collagen. Besides, it has good MS compatibility, and it is commonly used as embedding media for MALDI-MSI applications.^3,35,36^ In this work, we propose a tissue-like QCS by comparing the ionization efficiency of a small molecule in gelatin with tissue homogenates as ground truth. As a small molecule, propranolol was chosen due to its good solubility in gelatin solution. Moreover, propranolol has a good ionization efficiency by MALDI and other MS imaging techniques and has been broadly measured within brain, lung, and kidney tissues.^37–40^

Our study aims to use this new QCS to evaluate the variation caused by sample preparation and instrument performance, detect outlier slides, and assist the selection of computational batch effect correction methods.

## Experimental Section

### Materials and Reagents

Gelatin from porcine skin (G1890-500G, gel strength ∼300 g bloom, Type A), (±)-propranolol hydrochloride (≥99% (TLC), powder), taurocholic acid sodium salt hydrate (≥95% (TLC), powder), and 2,5-dihydroxybenzoic acid (2,5-DHB, 98% purity) were obtained from Sigma-Aldrich (Zwijndrecht, The Netherlands). ULC/MS-grade methanol (MeOH), HPLC-grade chloroform, and water were obtained from Biosolve (Valkenswaard, The Netherlands). Indium-tin-oxide (ITO) coated glass slides were obtained from Delta Technologies (Loveland, USA). Stable isotope labeled (r)-propranolol-*d*_7_ hydrochloride (98% purity) was obtained from CYMIT QUÍMICA, S.L. (Barcelona, Spain). Animal organs, including chicken liver and heart, were purchased from a local supermarket (Albert Heijn, Maastricht, The Netherlands), fresh goat liver was collected from a local slaughterhouse, and all animal organs were stored for a long time at −80 °C.

### Quality Control Standard Preparation

Preparation of tissue homogenates: chicken liver, chicken heart, and goat liver homogenates were prepared by a Precellys 24 Touch from Bertin Technologies (Aix-en-Provence, France) and 1.0 mm glass beads from BioSpec Products (Bartlesville, OK, USA). 100 μL of water was added to every 10 mg of animal tissue before homogenization, at 5000 rpm speed, 30 s shaking, and 30 s resting for 5 cycles. Frozen tissue blocks were made by transferring the tissue homogenates into a silicone mold of 1 × 1 × 1 mm^3^ and freezing under −80 °C overnight. 15% gelatin solution was used to embed the blocks in a tissue embedding mold (Truncated-T8, Polysciences, Warrington, USA), quickly freezing on dry ice for 60 s and later stored at −80 °C.

Different concentrations of gelatin solution (10, 20, 40, 80 mg/mL, also referring to 1%, 2%, 4%, 8% in w/v %) were prepared by dissolving gelatin powder in water and incubated in an Eppendorf ThermoMixer C (Eppendorf Vertrieb Deutschland GmbH, Wesseling-Berzdorf, Germany) with 300 rpm, at 37 °C until fully dissolved. Propranolol and propranolol-*d*_7_ (internal standard) solutions were individually prepared with water in 10 and 5 mM. QCS solution was prepared by mixing propranolol or propranolol-*d*_7_ solution with gelatin solution in a ratio of 1:20. A series of propranolol standard solutions were prepared at concentrations of 5, 4, 2.5, 2, 1.25, 0.5, 0.25, and 0.1 mM, used for pretesting the optimal gelatin concentration.

### Slide Preparation with Quality Control Standards

First, to evaluate QCS alone, a total of nine slides were prepared. The QCS solution was incubated at 37 °C for 30 min before spotting 18 1 μL spots onto an ITO slide, with spotting order randomized. Each slide thereby shared the same spotting pattern with 6 QCS spots per row and a total of 3 rows (Figure S1). Three slides with freshly spotted QCSs were prepared per day. After a 4 °C overnight drying step, slides were transferred to a vacuum desiccator for 30 min before matrix application. Rows per slide were measured sequentially within 1 day. A total of nine slides were measured: three per day for three consecutive days.

Second, to implement our QCS in routine MALDI-MSI workflows, six QCS spots were placed surrounding tissue regions. Tissue sections were sectioned at 12 μm thickness using a cryotome (CM1850, Leica Biosystems) and thaw-mounted on the ITO Slide, in the middle of the QCSs. All slides were stored at −80 °C until measurement. Each day, only slides to be measured on the same day were transferred to a vacuum desiccator for drying before matrix application. The preparation workflow can be seen in Figure 1.

**Figure 1:**
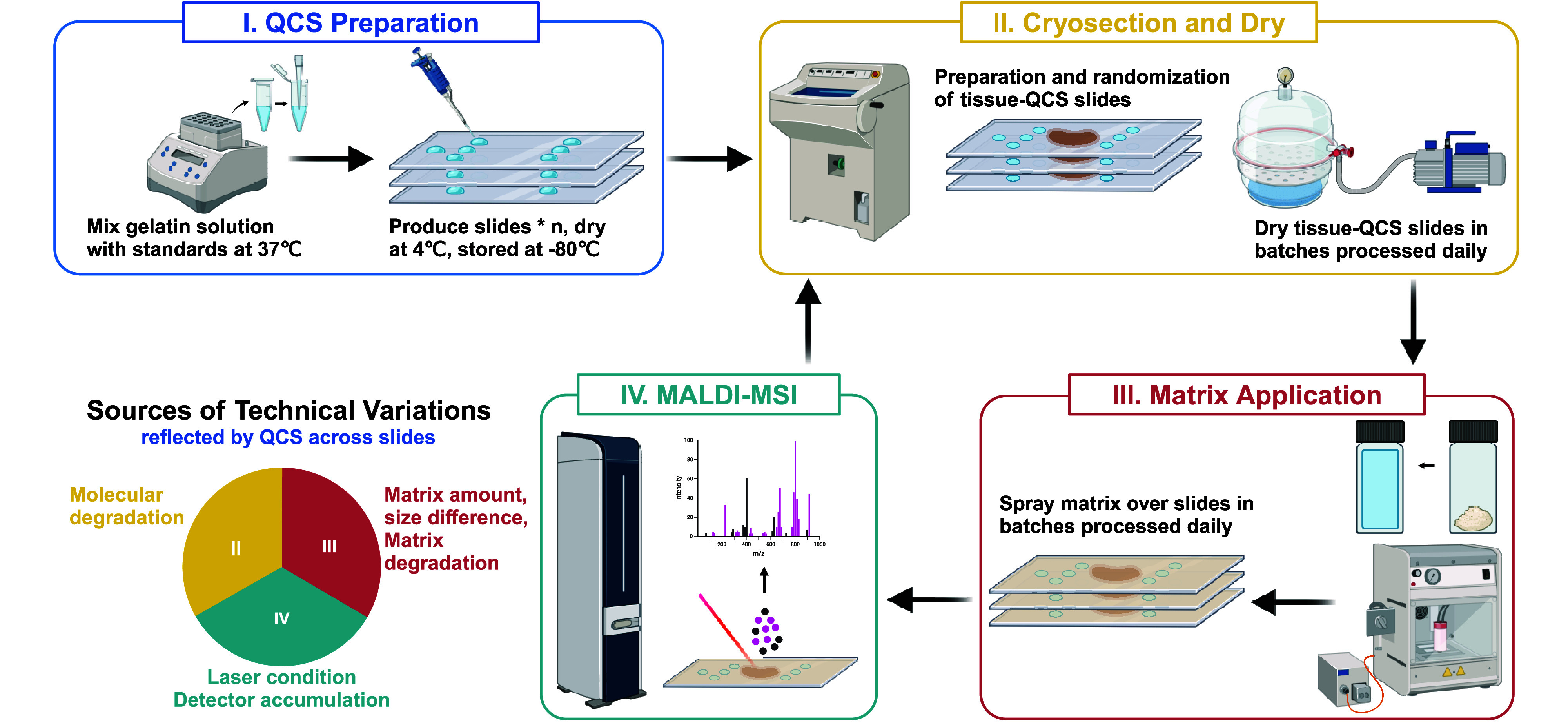
General QCS MALDI-MSI workflow. The technical variations introduced from every step are summarized on the bottom left. Molecular degradation resulting from temperature fluctuations can occur during the cryosection and dry stages (II). Variations in the matrix application (III) can involve errors occurring across slides, such as differences in the weighing of matrix, variations in crystal size, changes in sprayer conditions, and degradation of the matrix over time. During MALDI-MSI analysis (IV), laser conditions may vary and the accumulation of matrix at the detector can affect detection. This figure was produced using BioRender (https://BioRender.com/oz67vk1).

### Matrix Application

2,5-DHB (15 mg/mL) dissolved in chloroform/methanol (2/1, v/v) was selected as the coating matrix. Slides were coated with 2,5-DHB matrix using an HTX M3+ sprayer (HTX imaging LLC, Carrboro, NC, USA) with the following settings: temperature = 50 °C, flow rate = 120 μL/min, velocity = 1200 mm/min, track spacing = 3 mm, N_2_ gas pressure = 10 psi, drying time = 30 s, number of passes = 10, in a C–C pattern.^41^

### MALDI-MSI Data Acquisition

MALDI-MSI data acquisition was performed on a rapifleX MALDI Tissuetyper (Bruker Daltonik, Bremen, Germany) operated in the reflectron mode. Data were acquired in positive ion mode with a mass range of *m*/*z* 100–1000 and a pixel size of 100 × 100 μm. The laser frequency was set to 10 kHz, and 100 shots were accumulated at each pixel. Time-of-flight calibration was performed using red phosphorus before sample measurement.

### Data Processing and Analysis

SCiLS Lab MVS 2024b Premium 3D (SCiLS GmbH, Bruker, Bremen, Germany) was used for processing MALDI-MSI data. Raw data files from all sample slides in the batch test were together imported into one SCiLS file. The imported mass spectra were processed with baseline subtraction (deconvolution algorithm with a window width of 20). Peak picking was performed separately for tissue sections and QCS spots. Tissue feature finding worked on filtering 200 peaks in the mean spectrum, followed by excluding features irrelevant to tissue based on the generated feature ion images. QCS features including *m*/*z* of propranolol and propranolol-*d*_7_ were extracted from QCS spot regions. Final feature tables including QCS features and tissue features were exported in two formats: no normalization and TIC normalization. TIC normalization divides the peak abundance by the sum of all detected peaks. IS normalization was performed externally by dividing the peak abundance of propranolol by the abundance of its IS (propranolol-*d*_7_). The variation of QCS (propranolol) or tissue features was represented by coefficient variance (CV %), which was calculated by the standard deviation of a feature’s abundance divided by its mean abundance and reported as percentages.

Three open-source packages for batch effect correction were used, including Combat (R package of “sva”, version 3.19.0), WaveICA (R package of “WaveICA”, version 0.1.0), and NormAE (Python package of “NormAE”, https://github.com/luyiyun/NormAE.git). Combat uses an empirical Bayesian framework to model the linear batch effect, which was initially used in microarray data.^19^ WaveICA, developed for metabolomics, employs a matrix factorization technique combining a wavelet transform and independent component analysis to eliminate batch effects.^22^ NormAE, a deep neural network method also developed for metabolomics, utilizes autoencoder and adversarial learning techniques to address nonlinear batch effects without overfitting.^25^ For testing NormAE, we assigned all samples as one group in the input batch information to avoid correction in a supervised manner. Default method parameters were used when applying each package.

Corrected and raw data matrices were further evaluated in terms of propranolol and propranolol-*d*_7_ feature variations, tissue sample clustering, tissue feature variations, and differential biological feature quality. Principal component analysis (PCA) was performed with data centering and scaling to reduce data dimensionality and visualize the sample clustering within a 2D score plot. The Euclidean distance of QCSs within the sample group and between sample groups in the PCA score space was calculated. Average intragroup distance was calculated by averaging the straight-line distance of individual sample points from an intragroup centroid in the first and second principal component. The shorter intragroup distance indicates a higher sample proximity within the group. Likewise, pairwise distance calculation between each centroid of tissue groups was used to measure the average intergroup distance in the first and second principal component. The higher intergroup distance indicates a distinctive separation between the different sample types.

Partial least-squares discriminant analysis (PLS-DA) was used to identify differential features between different tissue types. Variable importance in the projection (VIP) of PLS-DA greater than 1.2 was considered as a distinctive feature.^42^ All statistical analyses and data visualizations were performed using R (version 4.2.2). Based on this, we also constructed a data pipeline for performing batch evaluation and correction with our QCSs step by step. The data pipeline is built in Jupyter Notebook with R programming language and is available on GitHub (https://github.com/Jintonic0226/QCS_Pipeline/tree/main).

## Results and Discussion

Establishing good quality controls is essential to any omics studies, given the rigorous demands for result reproducibility and maximizing MSI data quality. The use of alternative tissue-based controls in MSI analysis to evaluate batch effects remains limited in applicability and has been reported in only a few studies. In this work, we intend to propose a generic solution to capture the combined variations of an MSI workflow using QCSs. In this study, we have used the popular MALDI-MSI in which technical variation can be caused by the consecutive processes of cryosection, drying, matrix solution preparation, matrix spraying, and instrumental performance. To do so, we created tissue-mimic QCS using gelatin as a material imitating an ECM-like structure. Propranolol was selected to assess the ionization efficiency of a small molecule (mimicking endogenous metabolites) while avoiding biological variance. The complete design and layout are depicted in Figure 1.

### Quality Control Standard Fabrication

Our study first tested different gelatin concentrations (10, 20, 40 mg/mL, also referring to 1%, 2%, 4%) and compared with tissue homogenates (100 mg/mL) to select the best condition mimicking propranolol behavior. 50 μL of propranolol ranging from 0.10–5.0 mM, which is equivalent to 1.30–64.84 μg, was spiked into 500 μL of tissue homogenates, or 1%/2%/4% gelatin solution. The linear response of propranolol was built based on peak abundance with no normalization. Figure 2 shows that 4% gelatin resembled the liver tissue, in terms of ionization efficiency. The same conclusion could be also reached based on peak abundance with TIC normalization, shown in Figure S2. Therefore, we determined a final tissue-mimicking standard composed of 4% gelatin and propranolol, in an equivalent amount of 3.24 μg of propranolol per milligram of gelatin. Internal standard is well known for accounting for potential artifacts during slide preparation, analyte extraction, and ionization.^43,44^ The ratio of propranolol to its IS can be used as an internal check for QCS to determine the lower limit of observable variations by compensating for various potential sources of variability throughout the analytical process. Thus, propranolol-*d*_7_ was also spiked into QCS in an equivalent amount of 1.62 μg per milligram of gelatin. Additional experiments were performed to investigate the propranolol response by adding taurocholic acid to mimic the lipid composition in different gelatin concentrations. We created QCSs composed of propranolol alone in gelatin solution ranging from 2% to 4% to 8% and propranolol mixed with taurocholic acid (ratio in w/w is 1:4.15). This experiment was repeated across 3 days, with three technical replicates per day, and results are shown in Figure S3. With propranolol alone, 4% gelatin resembles the tissue response, showing no significant difference. The addition of taurocholic acid showed that 2% to 4% gelatin resembles propranolol response in tissue. Overall, 4% gelatin mimics the closest propranolol response in tissue regardless of the presence of taurocholic acid. However, adding taurocholic acid significantly increases the variability of propranolol detection across days (shown in Table S1). This type of additional variability would make batch effect evaluation more challenging. Therefore, to show our QCS pipeline as a proof of concept for small molecule MSI correction, we decided to use a single standard of propranolol.

**Figure 2:**
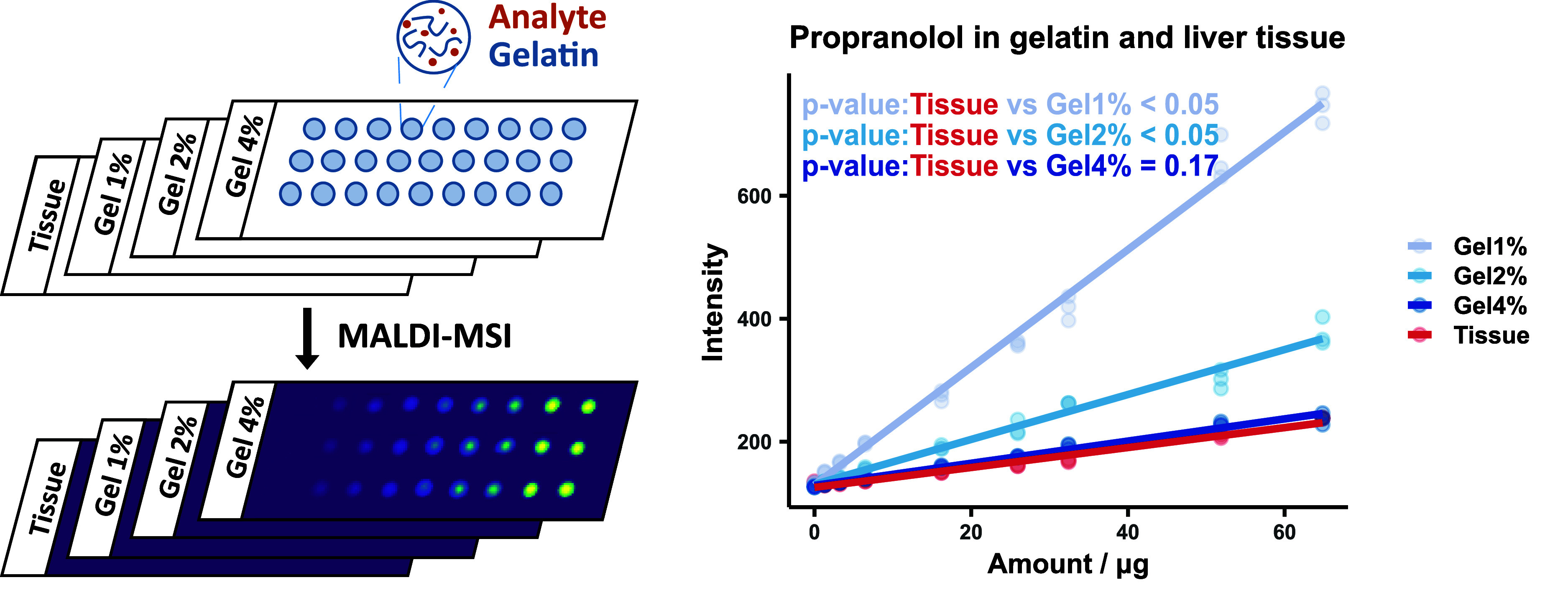
Evaluation of propranolol response in gelatin. Propranolol intensity is represented the *y*-axis, and propranolol amount spiked in different materials is represented in the *x*-axis. The propranolol amounts in serials are 1.30, 3.24, 6.48, 16.21, 25.93, 32.42, 51.87, and 64.84 μg. This is equivalent to 0.03–1.3 μg of propranolol per mg of tissue or 0.26–12.97/0.13–6.48/0.06–3.24 μg of propranolol per mg of gelatin (1%/2%/4%). The *R* ^2^ coefficient of the linear regression model for 1% gelatin (Gel1%) base is 0.99, for 2% gelatin (Gel2%) base is 0.98, for 4% gelatin (Gel4%) is 0.98, and for tissue is 0.98. Labels with *p*-values based on ANOVA are shown.

### Technical Variation in QCS on Different Batch Levels

We tested the performance of our QCS in batches processed daily. Over 3 days, a total of nine replicate slides were tested, with three slides freshly prepared and processed per day (Figure S1). Each day, three slides were sprayed together with matrix and stored in a vacuum before measurement. Figure 3A shows intraday CV % showing a decreased trend from 38.05% on day 1 to 17.89% on day 3. TIC normalization reduced the interday CV % from 32.01% to 25.96% and partially reduced the intraday CV % for days 1 and 2. No ion source cleaning was carried out between slide analyses except for a routine cleaning before or after the entire batch test. Using IS normalization, both intra- and interday variations were significantly reduced to below 12%. This is important, since a CV of 15% is generally regarded as the upper limit for analyte variability in bioanalytical method validation.^45^ Figure S4 further illustrates the QCS variability by rows within and across the slides. In addition, QCS variations within one slide can be observed in Figure 3B. Overall, raw QCS data preserved the technical variations during batch analysis and TIC normalization reflected a limited correction effect. IS normalization further represented a lower variation derived from matrix spraying, matrix degradation, ionization efficiency, and detector performance, thereby meeting the method validation criterion. Our results suggested good reproducibility in the fabrication and measurement of QCSs on slides using the current method.

**Figure 3:**
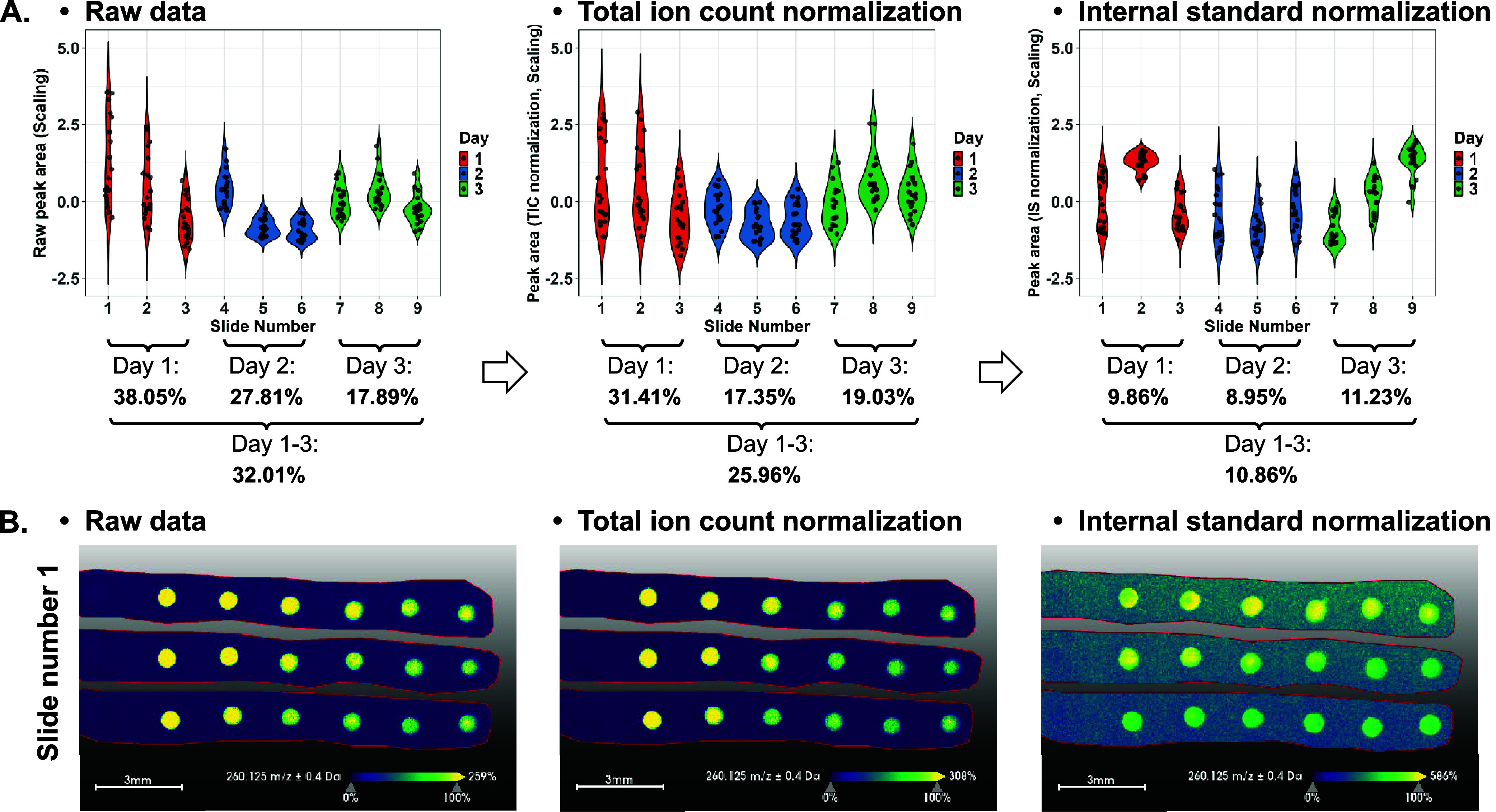
Batch effect evaluation of QCSs on ITO slides. (A) The *z*-scored peak area of propranolol measured by slides was visualized in violin plots, including raw data, TIC normalized data, and IS normalized data. Each slide contained six QCSs per row and a total of three rows. Intraday and interday variations (CV %) were shown below the violin plot. (B) Visualizations of propranolol on slide number 1 from day 1, presented as raw data, TIC normalized data, and IS normalized data. The scale bar shown in ion images is 3 mm.

### Batch Effect Evaluation and Correction of QCS

Next, we demonstrated the positive effect of our QCS with real tissues and its capability for batch effect reduction coupled with our developed pipeline (Figure 1). Three types of animal organs (goat liver, chicken liver, and chicken heart) were homogenized and frozen into separate blocks and further sectioned into replicates. Six replicate sections per organ were prepared on individual ITO slides with QCSs. For the effectiveness of quality control for MALDI-MSI, we proposed a layout, in which QCSs are placed surrounding the tissue section to capture intraslide spatial variability. Figure 4A illustrates the measurement layout, where MSI data acquisition is performed from left to right. Serial analysis was carried out in 3 days (six slides per day). Slide measurements were randomized to reduce the batch effect on further data analysis.^13^ Data visualization of the QCS and tissue sections is depicted in Figure S5. Raw data CV % on day 1 was abnormally high caused by slides 4–6 detected at low levels (Figure 4B, Table 1). This result might be related to the sprayer performance. Besides, the Kruskal–Wallis test revealed significant variations in QCS intensity both within day 1 (intraday1: *p* = 2.4 × 10^–5^) and between days (interday: *p* = 0.021), underscoring the need for correction. In comparison to raw data, TIC normalization on QCSs had a minor effect on reducing the interday CV % change. In contrast, IS normalization effectively corrected for the matrix impact and reduced the interday CV % to 14.11%. Figure S6 further shows a decrease in the intensity mean of QCSs after tissue measurements from the same slide. This decrease could be reduced through TIC and IS normalization.

**Figure 4:**
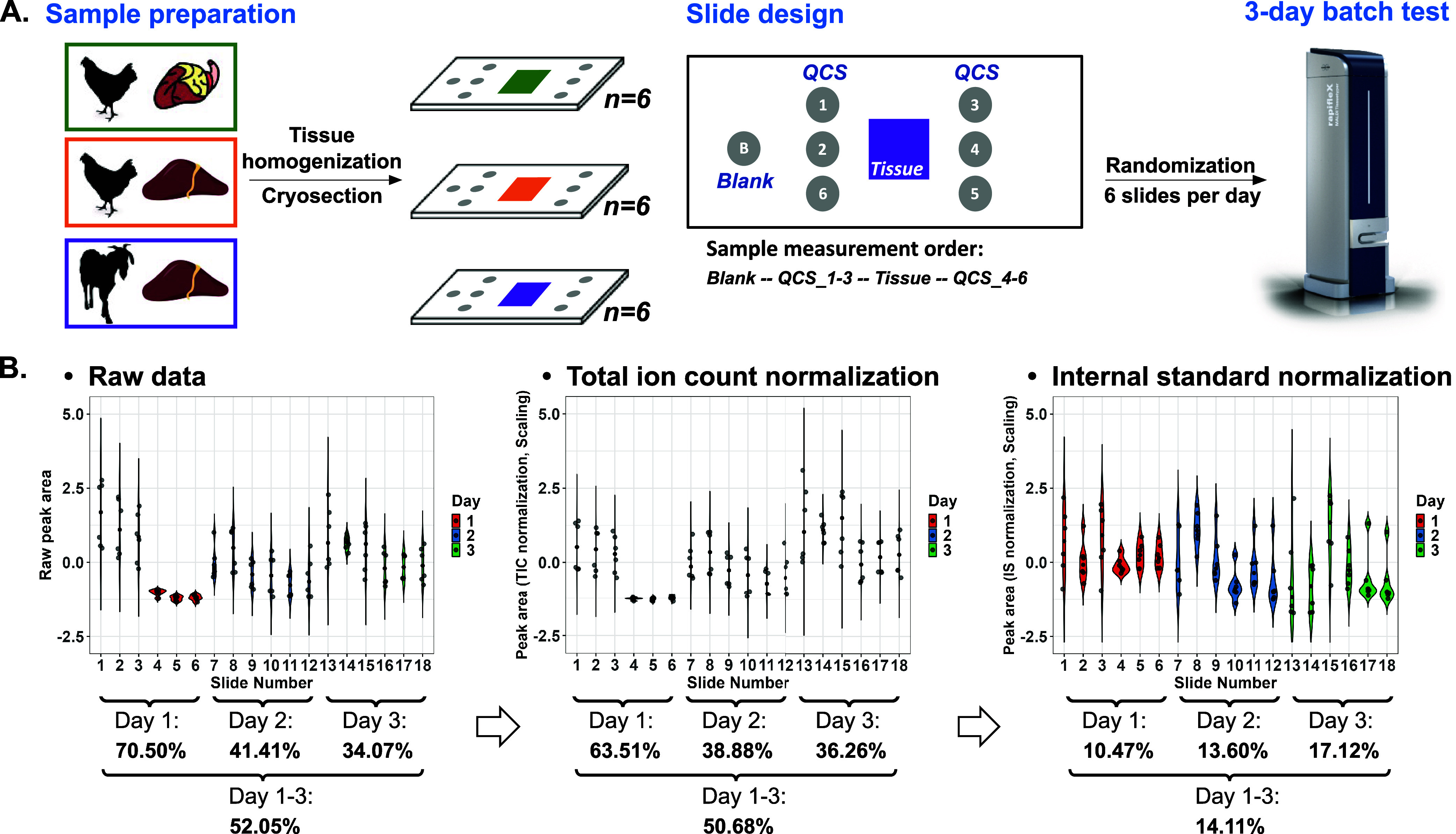
Evaluation of QCSs for batch tissue analysis. (A) Routine MSI workflow for batch effect evaluation. (B) The *z*-scored peak area of propranolol measured by slides was visualized in violin plots, including raw data, TIC normalized data, and IS normalized data. Each slide contained six QCSs. Intraday and interday variations (CV %) were shown below the violin plot. The used animal and organ image vectors were downloaded from VectorStock (https://www.vectorstock.com/).

**Table 1:** QCS Univariate Variation and Proximity Comparison before and after Batch Effect Correction^a^

method	intraday1 CV %	intraday2 CV %	intraday3 CV %	interday CV %	distance in PCA
raw	70.50	41.41	34.07	52.05	2.57
TIC	63.51	38.88	36.26	50.68	0.56
IS	10.47	13.60	17.12	14.11	na
TIC + Combat	55.34	43.41	35.21	46.02	0.51
TIC + WaveICA	26.98	30.70	37.46	32.49	0.62
TIC + NormAE	26.08	30.58	29.37	28.45	0.34
Combat	57.82	48.44	38.62	48.24	1.41
WaveICA	42.24	33.07	35.51	36.97	1.50
NormAE	24.79	25.70	23.21	24.37	0.89

a Label of “na” indicates no available data due to non-applicability.

However, for MALDI-MSI applications in metabolomics, the incorporation of an IS is not always feasible due to costs and complexity when analyzing several compounds or endogenous biomolecules in one single experiment. Therefore, for bioapplications, we incorporated further correction of batch effects using different open-source software, including Combat,^19^ WaveICA,^22^ and NormAE.^25^ Both QC samples and tissue samples were processed equally in an unsupervised manner. The correction effect on QCSs was used as a major quantitative metric to evaluate the software performance. QCS data normalized with IS were used as a reference for comparing the batch effect correction with other normalization or correction methods.

With the applied batch effect correction packages shown in Figure S7, QCSs on the outlier slides with low propranolol intensities (No. 4–No. 6) due to matrix impact were better corrected with WaveICA and NormAE compared to Combat. Table 1 summarizes the QCS interday CV % reached 24.37% with only NormAE, 28.45% with TIC and NormAE, and 32.49% with TIC and WaveICA. The average QCS distance in the PCA score plot (Figure S8) reflects the proximity of QCS in terms of all detected features, including propranolol and propranolol-*d*_7_, and tissue biological features present at a baseline level in QCS regions. The lowest distance was 0.34 achieved with TIC and NormAE. Generally, the correction packages combined with TIC normalization showed a better correction effect on QCS, bringing the intraday and interday CV % closer to IS normalization.

### Batch Effect Evaluation and Correction of Tissues

Our second evaluation focused on the effectiveness of QCS coupled to data batch correction for tissue assessment. The benefit of computational batch effect correction was evaluated by measuring the distances of the tissues and their group centroids in the PCA space and the detected feature variations across batches. As shown in the PCA score plot of Figure 5A, the first 3 slides (S1, S2, S3) deviated from their correspondent group in the raw dataset. TIC normalization was able to significantly correct the MS detection difference on slides 1–3, reflected in better tissue type clustering and reduced intragroup distances. Despite the low QCS signals detected in slides 4 to 6, the corresponding tissue samples maintained good intragroup clustering, both with and without TIC normalization. Figure S9 further illustrates the MALDI-MS images generated from the first three principal components obtained via PCA of the TIC-normalized dataset. On top of TIC normalization, NormAE and Combat showed larger improvements in intragroup clustering than WaveICA (Figure 5B). Besides, the larger the pairwise group distance, the better group distinction could be achieved. Table S2 shows that normalization with TIC and NormAE achieved the largest group separations. Overall, the correction packages combined with TIC normalization showed a better correction effect on tissue as well as for QCS. With an observation of individual tissue feature variation in boxplots of Figure 5A and Table S3, TIC normalization had a significant effect on reducing the median CV % to below 20%. In agreement with PCA clustering, all three algorithms further improved TIC normalization, with NormAE reducing the median CV % to below 10%.

**Figure 5:**
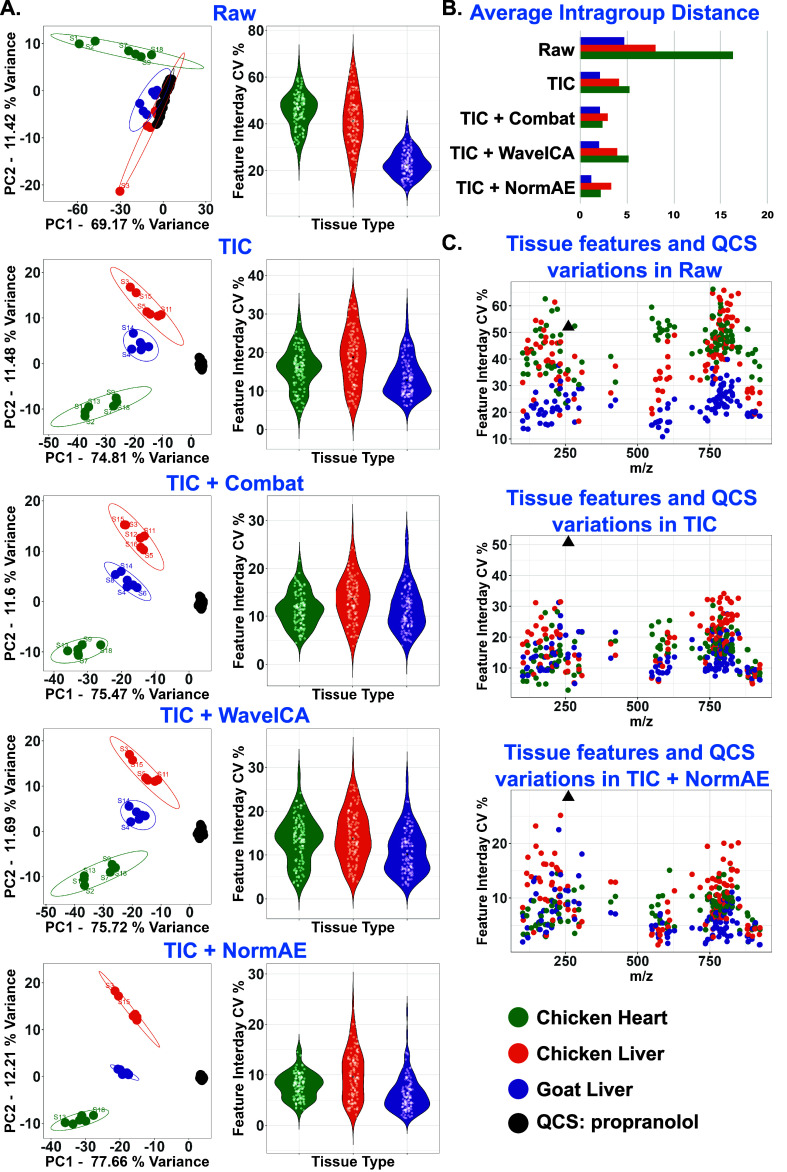
Comparisons of applying different batch effect correction methods for tissue classification. (A) PCA score plot (left) for QCSs and tissue samples measured from 3 batches and variation plot for metabolite features detected in tissue (right). The slide number was indicated by S1–S18. (B) Correspondent average intragroup distance in a bar plot. (C) Variation plot for metabolite features and QCS before correction and after correction with “TIC” and “TIC + NormAE” method.

By comparing the correction effect on tissue features with the QCS feature shown in Figure 5C, TIC normalization showed a limited effect on QCS correction due to a simplified QCS molecular composition. For tissue samples, TIC normalization showed good efficacy in correcting tissue feature variability across measurements. Overall, combining TIC and NormAE showed the best correction performance for both tissue features and QCS features. It is interestingly noted that compared to QCS, tissue features always exhibited more robustness against technical variance before and after any correction (Figure 5C), which could be a result of the complex tissue microenvironment and molecular composition. In contrast, our QCS showed higher sensitivity to technical variances, making them an effective indicator for evaluating batch effects before and after correction.

As demonstrated so far, the application of batch effect removal algorithms has led to closer replicates and smaller coefficients of variation. This could reflect the effective removal of unwanted experimental variations to a certain extent^46^ and thereby aid in the unmasking of previously undetected biological effects or the disproval of previously detected but false-positive observations. We hence investigated the correction effectiveness using supervised tissue classification in the form of PLS-DA. Distinct tissue features with variable importance scores (VIP) above 1.2 are summarized in Table S4. Generally, the number of distinct features decreased after batch effect correction, with five features being excluded following any correction. Ten features showed robustness, maintaining consistently high VIP scores across different correction methods as well as without correction. Figure S10 illustrates the intensity changes of two robust features relative to the measurement order. Lipid features (*m*/*z* > 700) were more subject to technical variations and could be identified as significant only after batch correction. Figure S11 illustrates the intensity changes of two lipids across the measurement order. Different batch correction methods mitigated the decline in lipid peak intensity over measurement order, thereby improving the separation between tissue types.

Finally, we showed a comprehensive evaluation of batch effect correction with the assistance of our designed QCS over a metabolomic dataset collected by MALDI-MSI. The ultimate selection of the batch effect correction method will still depend on specific research questions. A summary covering all investigated aspects in our study can be found in Table S5. Notably, our designed QCS is developed to account for batch effects in MSI analysis, with MALDI-MSI serving as an example.

This can specifically benefit the omics analyses across tissue samples in a mid- or large-scale study. To further minimize the variation derived from preparing QCSs, future improvements can be focused on the automated production of QCSs on the slides. Besides, new molecule species can be tested and incorporated to better mimic the tissue molecular compositions, for example, high MW proteins or peptides. The gelatin amount used in QCSs can be further tailored for different molecular compositions and tissue types.

Challenges still exist for handling the pixel-level relevant batch effect in the target sample. With a focus more on molecular changes across tissue regions within the target section, spraying a multiclass IS mixture over the section was recently demonstrated as a good approach.^43^

### User-Friendly Batch Evaluation and Correction Pipeline

In order to readily apply the QCSs workflow, we developed a companion computational pipeline coupled to our QCSs and provide excellent data analysis capabilities including monitoring of batch data quality, correction of batch effects, and pre- and post-evaluation. The pipeline contains two parts, as described in Figure 6. Part 1 focuses on evaluating the intrabatch and interbatch variations via QCS samples. The calculations of intrabatch and interbatch variations are displayed in a table or visualized in various plots. The results help to determine any slide outliers or the existence of issues with sample preparation or measurement before moving on to Part 2. Part 2 focuses on applying various batch effect correction methods and assessing the effectiveness of corrections via both QCS samples and tissue samples. Evaluations were conducted on data matrices before and after correction. The evaluation includes assessing the intrabatch and interbatch variations of standard or tissue features, using the Euclidean distance to measure the proximity of samples in the PCA score plot. Each part comprises data preprocessing, calculation and analysis, visualization, results comparison, and output generation.

**Figure 6:**
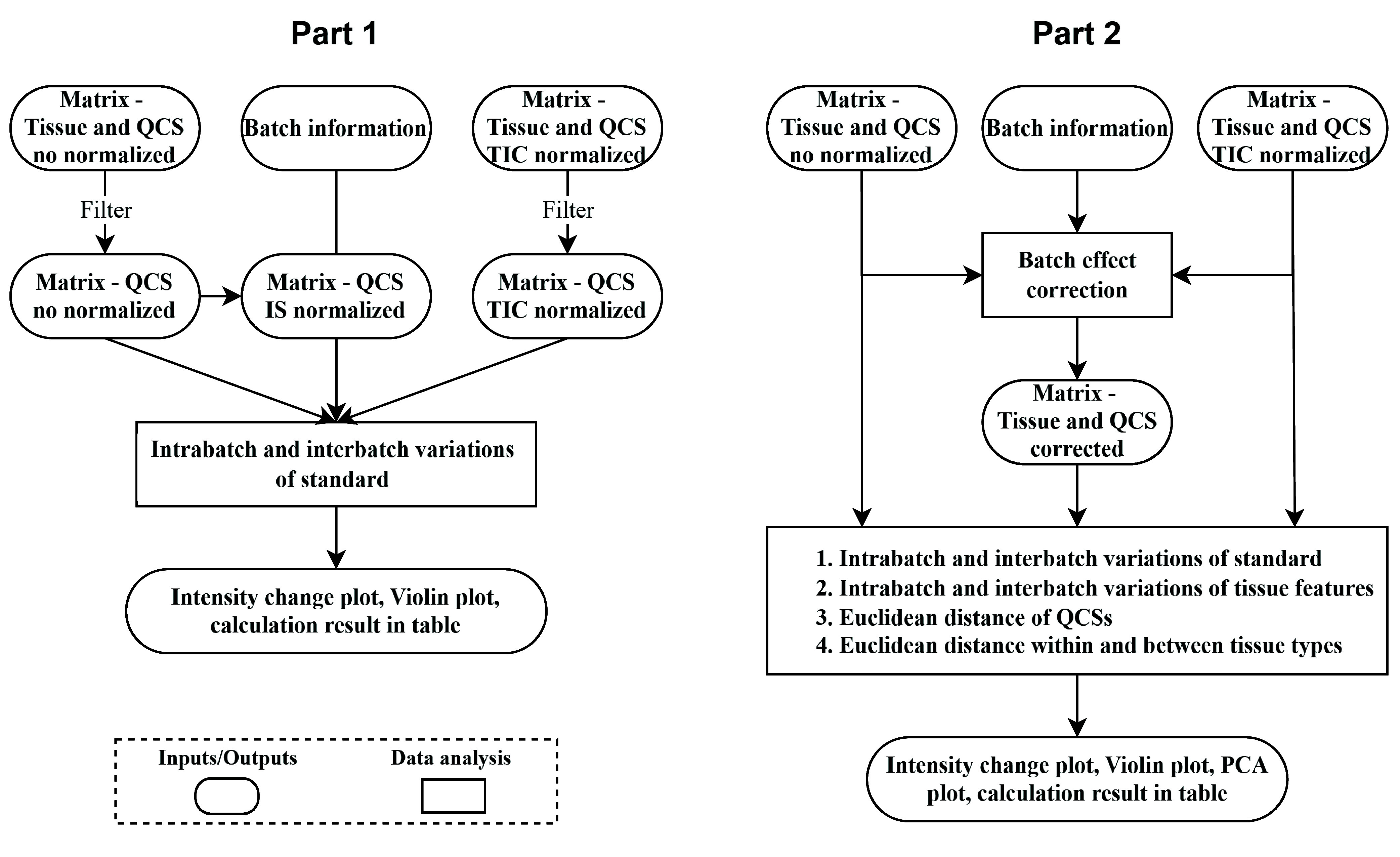
Illustration of working procedures of the data pipeline. Part 1 and part 2 utilize the same input data, including data matrices of tissue samples and QCSs with no or TIC normalization and batch information.

Current version offers human-readable and well-formatted source codes in the Jupyter Notebook. It allows the flexibility for users to easily modify parameters or incorporate new correction methods into the pipeline. Overall, our data pipeline helps users streamline data analysis with our QCSs application in the MALDI-MSI workflow. It uniquely combines batch effect evaluation and correction in one pipeline for MALDI-MSI data. It takes advantage of the recent advances in batch effect correction from the omics field. The pipeline requires a certain understanding of the programming language and could be further improved to give a more interactive interface.

## Conclusions

We designed and successfully applied reproducible and cost-effective gelatin-based QCS for MALDI-MSI workflows. Users can utilize the QCS data to evaluate the total variance of sample preparation and instrument performance. Besides, our QCSs pipeline can help in detecting outlier slides and assist in batch effect correction with an open-source and user-friendly data pipeline. The application of QCSs can be broad not only for inner lab experiments but also for multisite studies and will be particularly beneficial for moderate- or large-scale experiments.

## Supplementary Material



## Notes

Additional figures illustrating the experimental batch design; evaluation of QCS and tissue feature intensity variations with and without batch effect correction; MALDI-MS images; and tables summarizing the effects of different correction methods on QCS and tissue sample clustering and feature variation (PDF)
