# Supplementary material for: Quality Control Standards for Batch Effect Evaluation and Correction in Mass Spectrometry Imaging
Source: Anal Chem. 2025 May 12;97(20):10919–28. doi: 10.1021/acs.analchem.5c02020 (PMC12120824; doi:10.1021/acs.analchem.5c02020)
Supplement: Supplementary file 1 [file ac5c02020_si_001.pdf]

Quality Control Standards for Batch Effect Evaluation and Correction in Mass Spectrometry Imaging

Luojiao Huang[1], Yaejin Kim[1], Benjamin Balluff[2], Berta Cillero-Pastor[1,2,*]
[1] Cell Biology-Inspired Tissue Engineering, Institute for Technology-Inspired Regenerative Medicine, Maastricht University, 6229ER, Maastricht, Netherlands
[2] Maastricht MultiModal Molecular Imaging Institute, Division of Imaging Mass Spectrometry, Maastricht University, 6229ER, Maastricht, Netherlands
* Corresponding author: Berta Cillero-Pastor
Email: b.cilleropastor@maastrichtuniversity.nl

Supporting information includes:

Figure S1: Slide and batch design for measuring QCSs with MALDI-MSI.
Figure S2: Evaluation of different gelatin concentrations compared to liver homogenates in terms of propranolol detection.
Figure S3: Evaluation of propranolol (Pro) response with the addition of taurocholic acid (TCA) in gelatin.
Figure S4: Batch effect evaluation of QCSs on ITO slides.
Figure S5: Feature visualization from QCS and tissue section on the same slide.
Figure S6: Batch effect evaluation of QCSs on ITO slides for batch tissue analysis.
Figure S7: Intensity of propranolol in every QCS is affected by the measurement order.
Figure S8: PCA score plots for QCSs measured from 3 batches.
Figure S9: MALDI-MS images generated using the first three principal components obtained via principal component analysis of the TIC-normalized dataset.
Figure S10: Intensity plot of two significant features using PLS-DA multivariate analysis.
Figure S11: Intensity plot of two significant lipids using PLS-DA multivariate analysis.

Table S1: Relative standard deviation (RSD) of propranolol over three days.
Table S2: Intergroup distance calculation based on the PCA score plot before and after batch effect correction.
Table S3: Individual tissue feature variation and proximity comparison before and after batch effect correction.
Table S4: Feature selection with VIP above 1.2 based on PLS-DA multivariate analysis for tissue clustering datasets with and without batch effect correction.
Table S5: Summary of different correction methods for analyzing the three-day metabolomics batch.

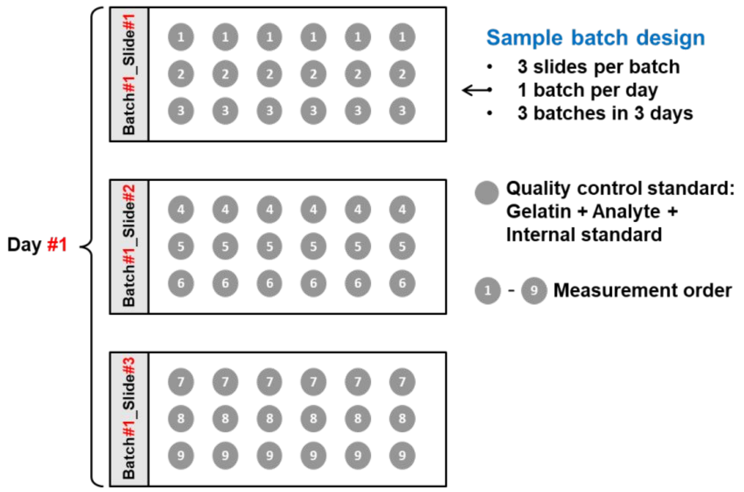

Figure S1. Slide and batch design for measuring QCSs with MALDI-MSI. Each slide has six quality control standards per row and three rows. Each row per slide was measured chronologically per day, three slides were measured per day, and three batches were measured in three days. The figure illustration shows the slide layout for day #1, day #2 and day #3 following the same pattern.

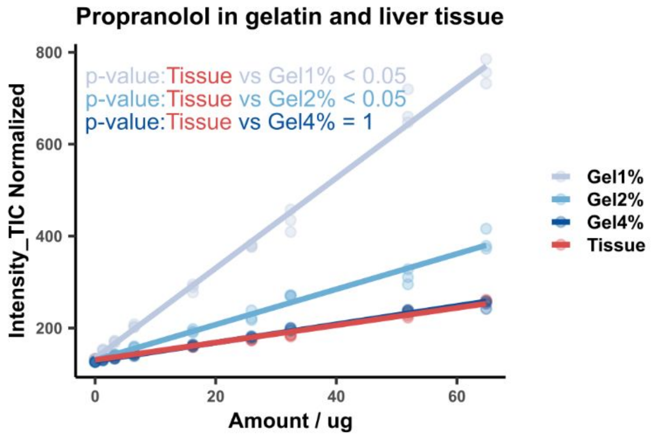

Figure S2. Evaluation of different gelatin concentrations compared to liver homogenates in terms of propranolol detection. Propranolol intensity after TIC normalization represented the y-axis, and the total amount of propranolol spiked in different materials represented the x-axis. The propranolol amounts in serials are 1.30, 3.24, 6.48, 16.21, 25.93, 32.42, 51.87 and 64.84 µg. This is equivalent to 0.03-1.3 µg of propranolol per mg of tissue, or 0.26-12.97 na 0.13-6.48 na 0.06-3.24 µg of propranolol per mg of gelatin (1% na 2% na 4%). The R^2 coefficient of the linear regression model for 1% gelatin (Gel1%) is *0.99*, for 2% gelatin (Gel2%) is $R^2=0.98$, for 4% gelatin (Gel4%) is $R^2=0.99$, and for tissue is $R^2=0.99$. Labels with p-values based on ANOVA analysis are shown.

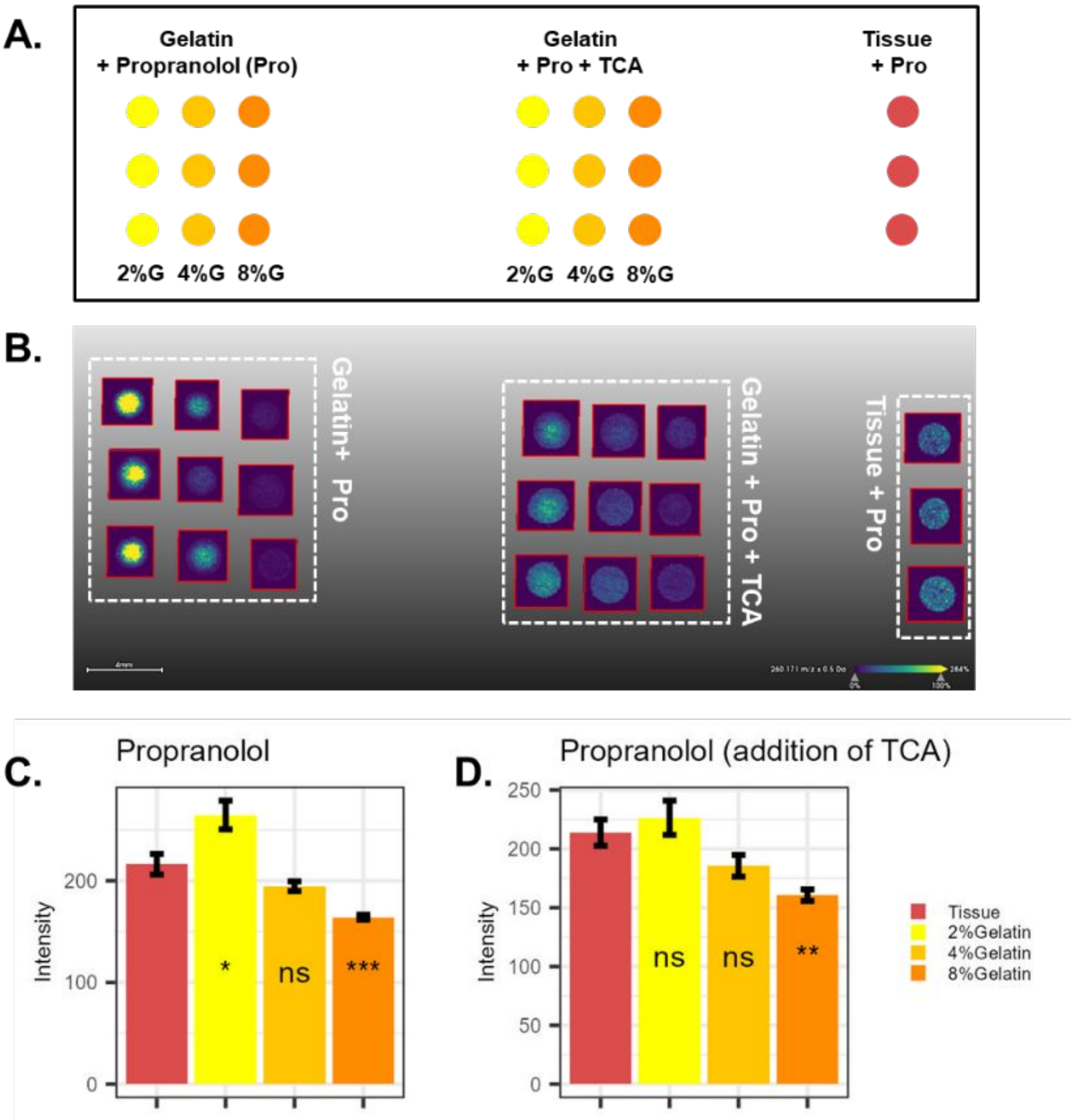

Figure S3. Evaluation of propranolol (Pro) response with the addition of taurocholic acid (TCA) in gelatin. Propranolol intensity is represented the y-axis, and propranolol amount spiked in tissue, 2%, 4% and 8% gelatin is equivalent to 0.41 µg/mg of propranolol in tissue, or 2.0 / 1.01 / 0.51 µg/mg of propranolol in gelatin (2% / 4% / 8%), TCA spiked in tissue, 2%, 4% and 8% gelatin is equivalent to 1.68 µg/mg of propranolol in tissue, or 8.40 / 4.20 / 2.10 µg/mg of propranolol in gelatin (2% / 4% / 8%). Labels representing statistical significance based on student t-test analysis are shown, ns: p-value > 0.05, *: p-values <= 0.05, **: p-values <= 0.01, ***: p-values <= 0.001.

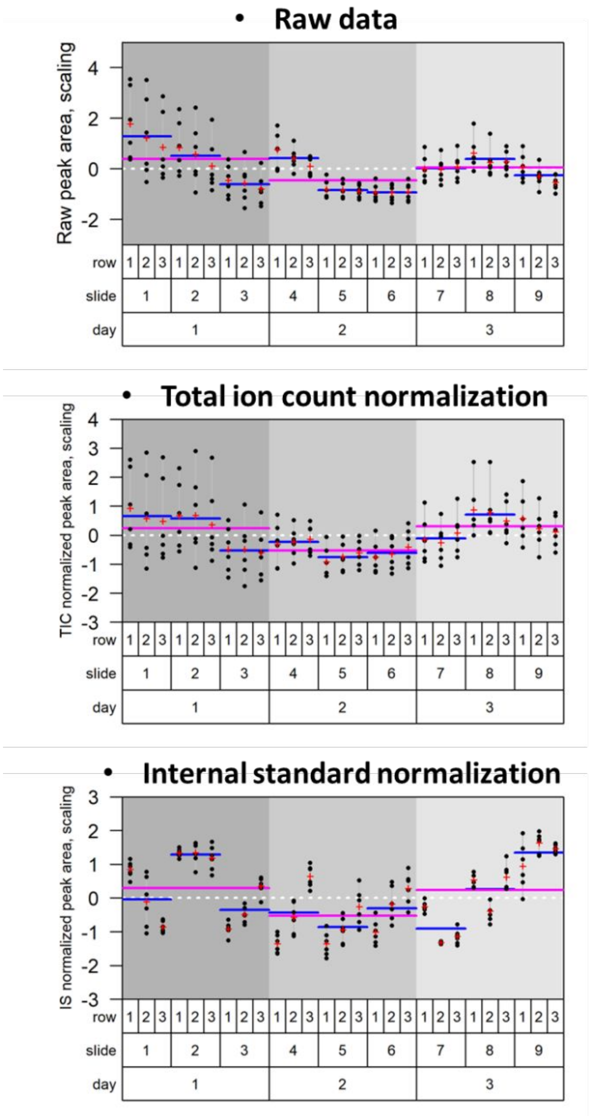

Figure S4. Batch effect evaluation of QCSs on ITO slides. The z-scored peak area of propranolol is visualized in a variability chart, including raw data, TIC normalized data, and IS normalized data. Each slide contained six QCSs per row and a total of three rows. + represents the mean value at a factor level of 'row'. The blue line indicates the mean value at a factor level of 'slide', the pink line indicates the mean value at a factor level of 'day', the white dashed line indicates the overall mean.

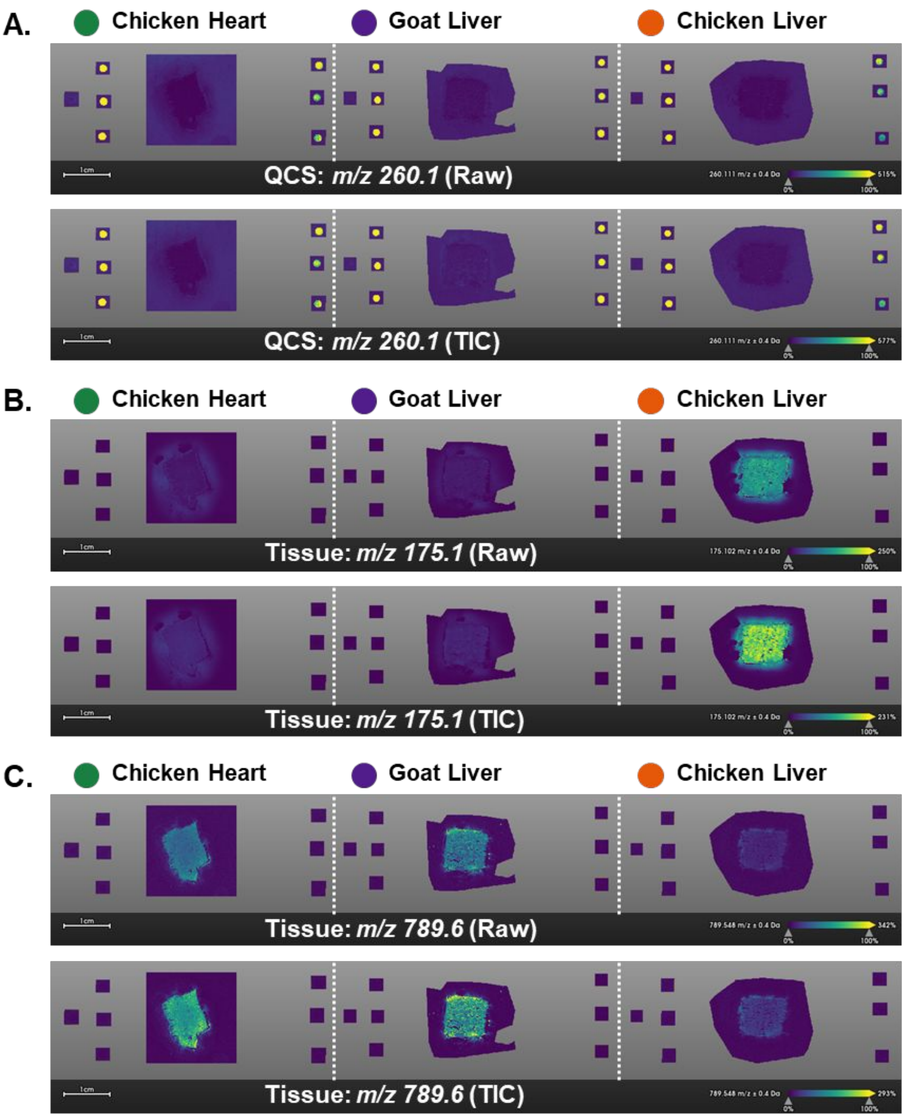

Figure S5. Feature visualization from QCS and tissue section on the same slide.

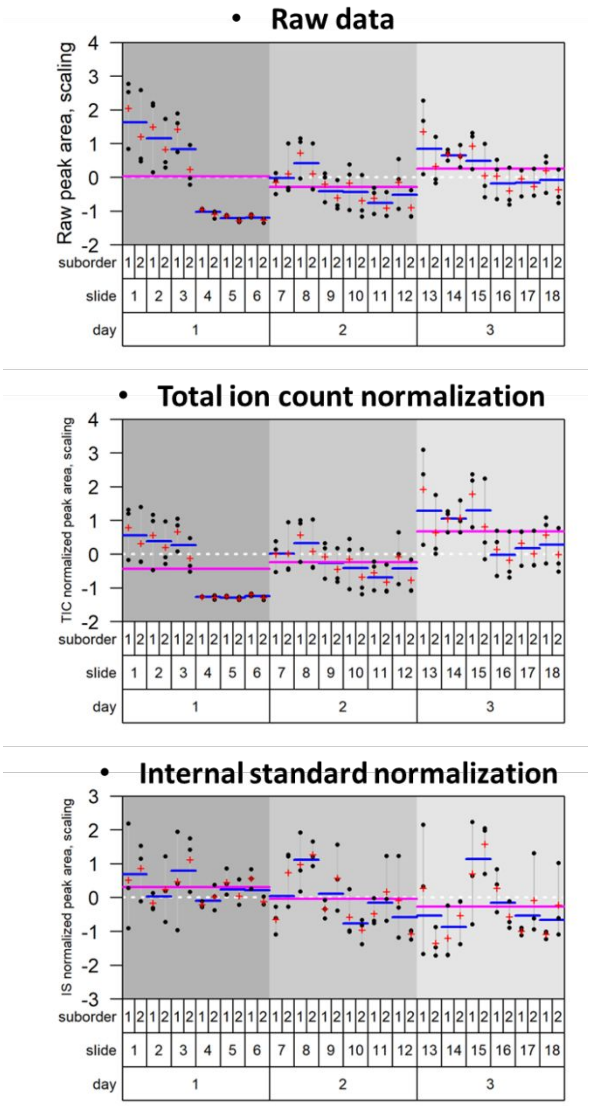

Figure S6. Batch effect evaluation of QCSs on ITO slides for batch tissue analysis. The z-scored peak area of propranolol is visualized in a variability chart, including raw data, TIC normalized data, and IS normalized data. Each slide contained six QCSs surrounding the tissue section. Among these, three QCSs were measured before the tissue section, and three were measured after the tissue section. + represents the mean value of three QCSs measured in the same suborder. The blue line indicates the mean value at a factor level of 'slide', the pink line indicates the mean value at a factor level of 'day', the white dashed line indicates the overall mean.

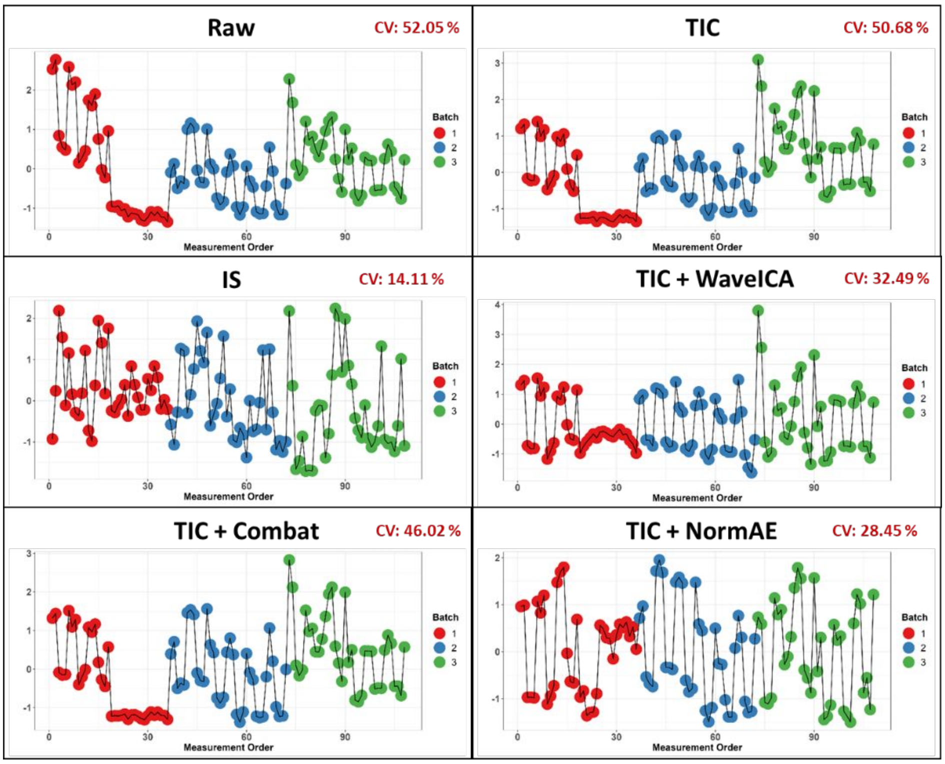

Figure S7. Intensity of propranolol in every QCS is affected by the measurement order. Each block shows intensity after scaling in raw or applied with normalization methods (TIC and IS), or batch effect correction methods (WaveICA, Combat, NormAE). Measurement number 24 to 36 corresponds to slide number 4 to 6.

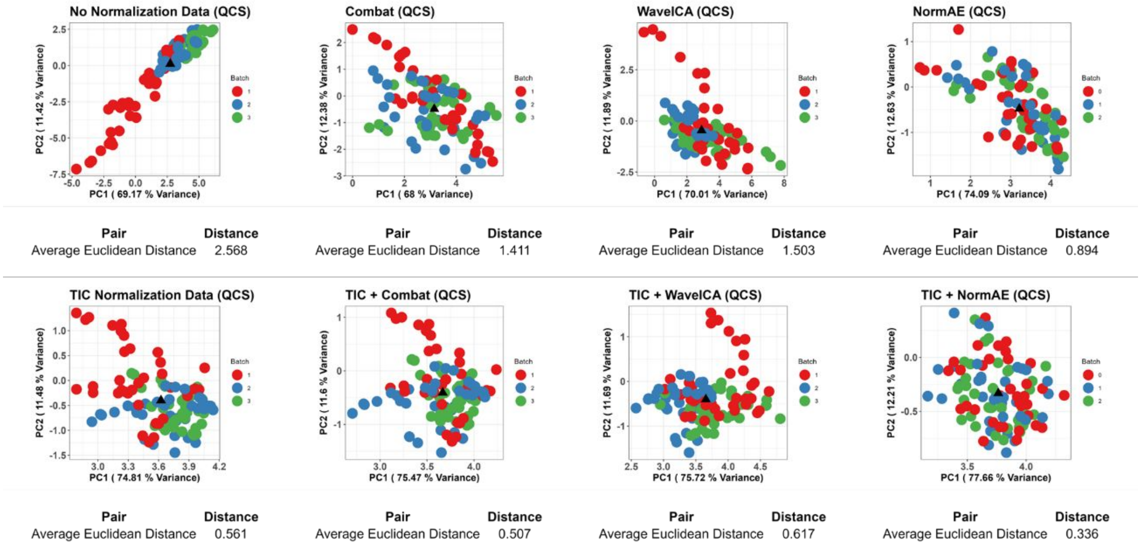

Figure S8. PCA score plots for QCSs measured from 3 batches. The average Euclidean Distance between QCSs based on the first two components is shown below each plot. The dot color annotates the batch order.

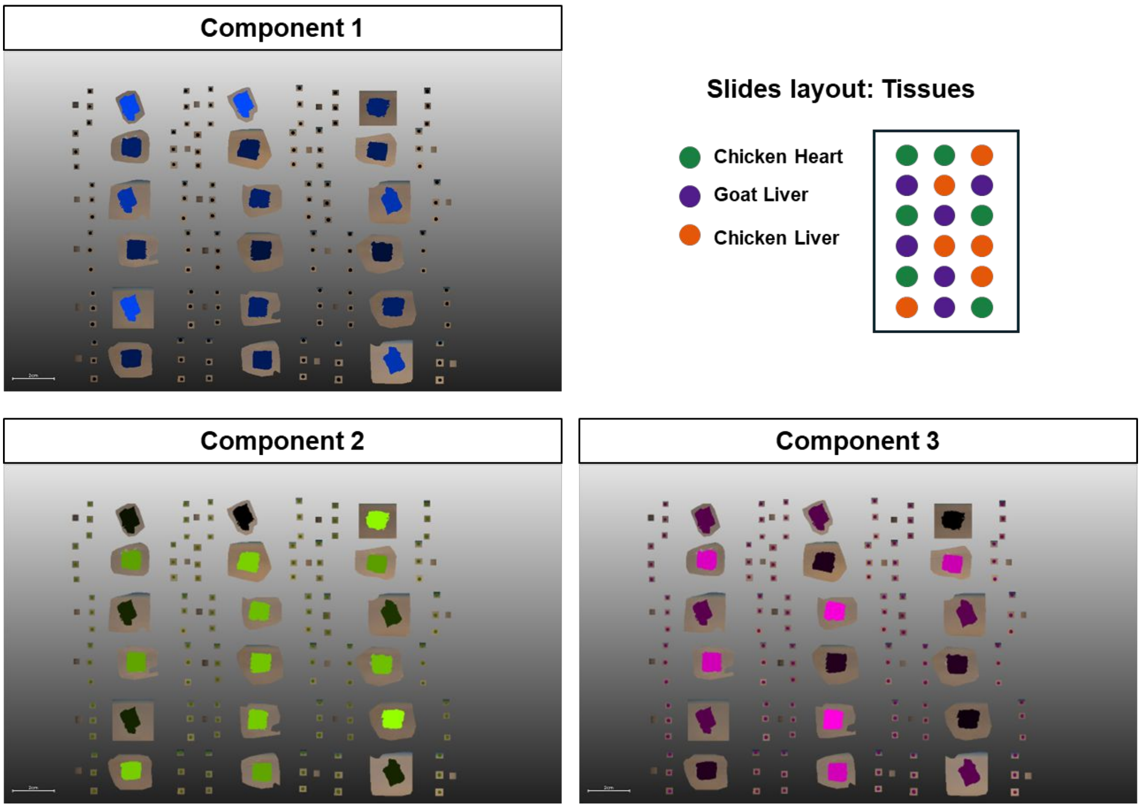

Figure S9. MALDI-MS images generated using the first three principal components obtained via principal component analysis of the TIC-normalized dataset.

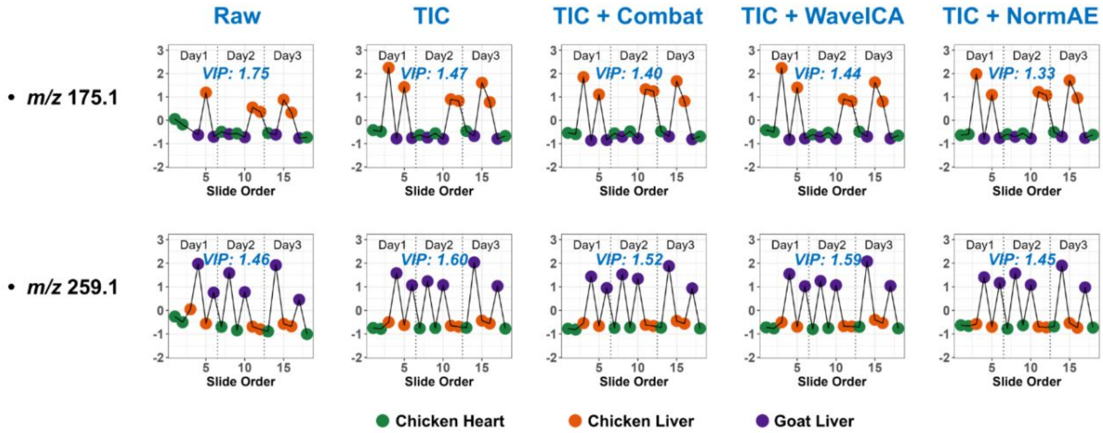

Figure S10. Intensity plot of two significant features using PLS-DA multivariate analysis. The feature intensity was compared between raw data and processed data with different batch correction methods. The y-axis represents the ion peak abundance after scaling. Features of VIPs above 1.2 were labeled in blue text and different tissue types are represented in different colors.

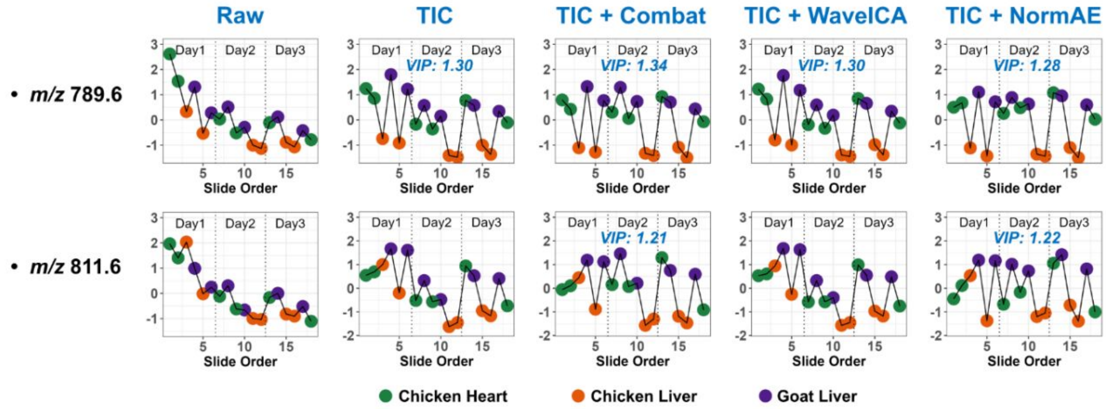

Figure S11. Intensity plot of two significant lipids using PLS-DA multivariate analysis. The feature intensity was compared between raw data and processed data with different batch correction methods. The y-axis represents the ion peak abundance after scaling. Features of VIPs above 1.2 were labeled in blue text and different tissue types are represented in different colors.

Table S1. Relative standard deviation (RSD) of propranolol over three days.

	a. Propranolol only	b. Propranolol_withTCA	Changes in RSD (b vs a)
Tissue	13.97%	15.61%	11.74%
2% Gelatin	15.96%	19.26%	20.68%
4% Gelatin	7.30%	14.95%	104.79%
8% Gelatin	4.31%	9.20%	113.46%

Table S2. Intergroup distance calculation based on the PCA score plot before and after batch effect correction.

	Chicken Heart - Chicken Liver	Chicken Heart - Goat Liver	Chicken Liver - Goat Liver	Average Intergroup Distance
**Raw**	25.00	21.95	6.19	17.71
**TIC**	27.42	19.30	8.85	18.52
**TIC + Combat**	27.86	19.32	9.21	18.80
**TIC + WaveICA**	27.63	18.71	9.80	18.71
**TIC + NormAE**	27.65	16.34	13.62	19.20

Table S3. Individual tissue feature variation and proximity comparison before and after batch effect correction.

	Intraday 1 CV%	Intraday 2 CV%	Intraday 3 CV%	Interday CV%	Distance in PCA
**Chicken Heart**					
**Raw**	12.84 ± 6.84%	16.93 ± 8.34%	26.88 ± 12.72%	45.32 ± 8.10%	16.26
**TIC normalization**	5.66 ± 4.95%	6.96 ± 5.03%	16.88 ± 10.24%	15.64 ± 5.59%	5.29
**TIC + Combat**	5.57 ± 4.96%	7.32 ± 5.38%	16.67 ± 10.19%	12.10 ± 5.51%	2.38
**TIC + WaveICA**	5.73 ± 5.50%	6.60 ± 7.24%	15.54 ± 9.81%	14.53 ± 10.12%	5.15
**TIC + NormAE**	5.11 ± 4.00%	6.03 ± 4.04%	12.50 ± 7.79%	8.17 ± 3.49%	2.24
**Chicken Liver**					
**Raw**	37.55 ± 10.32%	6.51 ± 5.53%	11.49 ± 7.60%	41.29 ± 11.85%	8.08
**TIC normalization**	13.51 ± 8.94%	6.67 ± 5.24%	12.02 ± 7.56%	18.61 ± 7.56%	4.18
**TIC + Combat**	13.28 ± 8.89%	6.93 ± 5.29%	11.87 ± 7.46%	14.11 ± 6.35%	2.93
**TIC + WaveICA**	12.19 ± 12.43%	5.16 ± 4.61%	11.30 ± 12.67%	15.55 ± 11.20%	3.98
**TIC + NormAE**	13.72 ± 8.71%	4.24 ± 3.16%	11.80 ± 7.28%	10.03 ± 5.01%	3.30
**Goat Liver**					
**Raw**	23.72 ± 8.48%	25.12 ± 7.45%	23.86 ± 9.95%	23.03 ± 5.61%	4.70
**TIC normalization**	8.68 ± 6.63%	9.15 ± 5.28%	10.27 ± 8.49%	13.41 ± 5.13%	2.11
**TIC + Combat**	8.52 ± 6.62%	9.59 ± 5.55%	10.14 ± 8.32%	12.11 ± 5.82%	2.14
**TIC + WaveICA**	8.01 ± 6.57%	7.34 ± 5.46%	11.11 ± 17.51%	11.91 ± 9.36%	2.06
**TIC + NormAE**	5.56 ± 4.62%	6.25 ± 4.44%	8.13 ± 6.96%	6.37 ± 3.70%	1.23

Table S4. Feature selection with VIP above 1.2 based on PLS-DA multivariate analysis for tissue clustering datasets with and without batch effect correction.

Feature *m/z*	Raw	TIC	TIC_Combat	TIC_WaveICA	TIC_normAE
**175.1**	1.75	1.47	1.40	1.44	1.33
**102.1**	1.75	1.43	1.36	1.32	na
**140.1**	1.73	1.41	1.33	1.34	na
**258.1**	1.72	1.67	1.58	1.63	1.50
**120.1**	1.70	1.34	1.28	1.32	1.24
**296.1**	1.68	1.64	1.55	1.62	1.50
**280.1**	1.65	1.63	1.54	1.62	1.49
**251.1**	1.63	1.63	1.54	1.61	1.48
**282.1**	1.62	1.31	1.25	1.29	na
**118.1**	1.60	1.22	na	1.22	na
**297.0**	1.54	1.61	1.52	1.60	1.49
**158.1**	1.52	1.27	1.22	1.25	na
**189.1**	1.47	1.22	na	1.22	na
**104.1**	1.46	1.25	na	1.25	na
**259.1**	1.46	1.60	1.52	1.59	1.45
**227.1**	1.45	1.54	1.47	1.53	1.41
**307.1**	1.44	1.45	1.38	1.42	1.31
**204.1**	1.44	na	na	na	na
**116.1**	1.42	na	na	na	na
**182.1**	1.33	na	na	na	na
**166.1**	1.29	na	na	na	na
**213.1**	1.28	na	na	na	na
**202.1**	1.20	1.25	na	1.23	na
**789.6**	na	1.30	1.34	1.30	1.28
**788.6**	na	1.20	1.26	1.21	na
**184.1**	na	na	1.22	na	na
**811.6**	na	na	1.21	na	1.22
**219.1**	na	na	na	1.21	na
**741.5**	na	na	na	na	1.28
**820.5**	na	na	na	na	1.22

* Label of 'na' indicates feature with VIP below 1.2, representing no significant importance in tissue differentiation.

Table S5. Summary of different correction methods for analyzing the three-day metabolomics batch.

	Raw	TIC	TIC_Combat	TIC_WaveICA	TIC_normAE
**QCS univariate variation** Interday CV%	52.05%	50.68%	46.02%	32.49%	28.45%
**QCS PCA proximity**	2.57	0.56	0.51	0.62	0.34
**Average tissue features variations in**					
Chicken Heart	45.32%	15.64%	12.10%	14.53%	8.17%
Chicken Liver	41.29%	18.61%	14.11%	15.55%	10.03%
Goat Liver	23.03%	13.41%	12.11%	11.91%	6.37%
**Tissue PCA proximity** between groups on average	17.71	18.52	18.80	18.71	19.20
**Tissue PCA proximity in**					
Chicken Heart	16.26	5.29	2.38	5.15	2.24
Chicken Liver	8.08	4.18	2.93	3.98	3.30
Goat Liver	4.70	2.11	2.14	2.06	1.23
**Number of distinct biological features**	23	20	18	21	14
